# Edge Detection in Digital Images Using Dispersive Phase Stretch Transform

**DOI:** 10.1155/2015/687819

**Published:** 2015-03-23

**Authors:** Mohammad H. Asghari, Bahram Jalali

**Affiliations:** ^1^Department of Electrical Engineering, University of California, Los Angeles, Los Angeles, CA 90095, USA; ^2^Department of Bioengineering, University of California, Los Angeles, Los Angeles, CA 90095, USA; ^3^Department of Surgery, David Geffen School of Medicine, University of California, Los Angeles, Los Angeles, CA 90095, USA

## Abstract

We describe a new computational approach to edge detection and its application to biomedical images. Our digital algorithm transforms the image by emulating the propagation of light through a physical medium with specific warped diffractive property. We show that the output phase of the transform reveals transitions in image intensity and can be used for edge detection.

## 1. Introduction

Edge detection is the name for a set of mathematical methods for identifying patterns in digital images where brightness or color changes abruptly [1–3]. Applying an edge detection algorithm to an image can be used for object detection and classification. It also reduces the digital file size while preserving important information, albeit data compression is not the main objective in edge detection.

Many methods for edge detection have been proposed, but most of them can be grouped into two main categories: zero-crossing based and search-based. The zero-crossing based methods search for zero crossings in a Laplacian or second-order derivative computed from the image [1]. The search-based methods compute the edge strength, usually with a first-order derivative, and then search for local directional maxima of the gradient amplitude [2]. Detailed survey of available techniques for edge detection can be found in [3].

We employ a physics-inspired digital image transformation that emulates propagation of electromagnetic waves through a diffractive medium with a dielectric function that has warped dispersive (frequency dependent) property. We show that the phase of the transform has properties conducive for detection of edges and sharp transitions in the image. Our method emulates diffraction using an all-pass phase filter with specific frequency dispersion dependencies. The output phase profile in spatial domain reveals variations in image intensity and when followed by thresholding and morphological postprocessing provides edge detection. We show how filters with linear and nonlinear phase derivatives can be used for edge detection and how the shape and magnitude of the phase function influence the edge image.

Earlier it was shown that the magnitude of the complex amplitude for a similarly transformed image exhibits reduction in space-bandwidth product and may be useful for data compression [4]. The present paper employs the phase of the transform for application to edge detection. Also, the details of the filter kernel are different in the two cases. Going further back, the concept of diffraction based image processing has its roots in the Photonic Time Stretch, a temporal signal processing technique that employs temporal dispersion to slow down, capture, and digitally process fast waveforms in real time [5]. Known as the time-stretch dispersive Fourier transform, this technique has led to the discovery of optical rogue waves and detection of cancer cells in blood with record sensitivity [6], as well as highest performance analog-to-digital conversion [7]. In this paper, we also demonstrate application of the proposed edge detection algorithm to some biomedical images.

## 2. Technical Description

**Figure 1:**
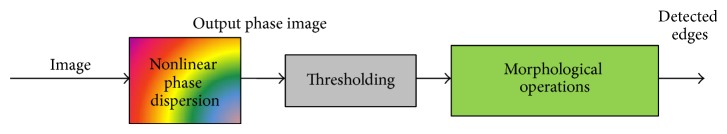
In the proposed method for edge detection, after the application of localization filter, a warped Phase Stretch Transform is performed on the image and the phase of the output image is thresholded and postprocessed by morphological operations to generate the image edges.

Different steps of the proposed method for edge detection are shown in Figure 1. In this method, the original image is first smoothed using a localization kernel and then is passed through a nonlinear frequency dependent (dispersive) phase operation, called Phase Stretch Transform (PST). PST applies a 2D phase function to the image in the frequency domain. The amount of phase applied to the image is frequency dependent; that is, a higher amount of phase is applied to higher frequency features of the image. Since image edges contain higher frequency features, PST emphasizes the edge information in the image by applying more phase to higher frequency features. Image edges can be extracted by thresholding the PST output phase image. After thresholding, the binary image is further processed by morphological operations to find the image edges.

In the remainder of this paper, we refer to this technique as the Phase Stretch Transform (PST). The image under analysis is represented by *B*[*n*, *m*] where *n* and *m* are two-dimensional spatial variables. The PST in frequency domain can be described as follows:

(1) An,m =∡IFFT2K~p,q·L~p,q·FFT2Bn,m,

where *A*[*n*, *m*] is the output phase image, *∡*〈·〉 is the angle operator,* FFT2* is the two-dimensional Fast Fourier Transform,* IFFT2* is the two-dimensional Inverse Fast Fourier Transform, and *p* and *q* are two-dimensional frequency variables. The function $\tilde{L}\left[p,q\right]$ is the frequency response of the localization kernel and the warped phase kernel $\tilde{K}\left[p,q\right]$ is described by a nonlinear frequency dependent phase:

(2) $$\begin{array}{c} \tilde{K}\left[p,q\right]=e^{j\cdot \varphi \left[p,q\right]}. \end{array}$$

While arbitrary phase kernels can be considered for PST operation, here we study the phase kernels for which the kernel phase derivative *PD*[*p*, *q*] is a linear or sublinear function with respect to frequency variables. A simple example of such phase derivative profiles (e.g., represented by least number of parameters) is the inverse tangent function which leads to the following PST kernel phase:

(3) φp,q=φpolarr,θ=φpolarr=S ·W·r·tan−1W·r−1/2·ln⁡1+W·r2W·rmax⁡·tan−1W·rmax⁡−(1/2)·ln⁡1+W·rmax⁡2,

where $r=\sqrt{p^{2}+q^{2}}$, *θ* = tan^−1^(*q*/*p*), tan^−1^(·) is the inverse tangent function, ln⁡(·) is the natural logarithm, and *r*_max⁡_ is the maximum frequency *r*. *S* and *W* are real-valued numbers related to the strength (*S*) and warp (*W*) of the phase profile applied to the image. For simplicity, we have assumed that the PST kernel phase profile has circular symmetry with respect to frequency variables. For small warping factors *W* ≪ 1, the phase profile *φ*[*p*, *q*] becomes a quadratic phase and represents the case with linear phase derivative. The two parameters *S* and *W* along with the width of the localization kernel and the thresholding values are used to extract the edge information.

Application of PST to the image creates the phase image *A*[*n*, *m*] which is further postprocessed. For edge detection, postprocessing includes cutting the negative phase values, thresholding, and morphological operations. Here we use one-level thresholding. Dependent on the application, the threshold can be set to allow more or less edges to be shown in the binary edge image. Morphological operations can be used to thin the edges, clean the phase image by removing the isolated pixels, or find prime lines representing edges. Frequency bandwidth (full width at half maximum) of the localization kernel is designed to reduce the noise in the proposed edge detection algorithm while preserving the vital edge information. In this paper we have used a Gaussian localization filter.

The parameters that are required to be designed for the proposed edge detection methods are

(4) (1)  S  and  W:  Strength  (S)  and  Warp  (W)    of  the  applied  phase  kernel(2)  Δf:  Bandwidth  of  the  localization  kernel(3)  Thresh:  Threshold  value.

**Figure 2:**
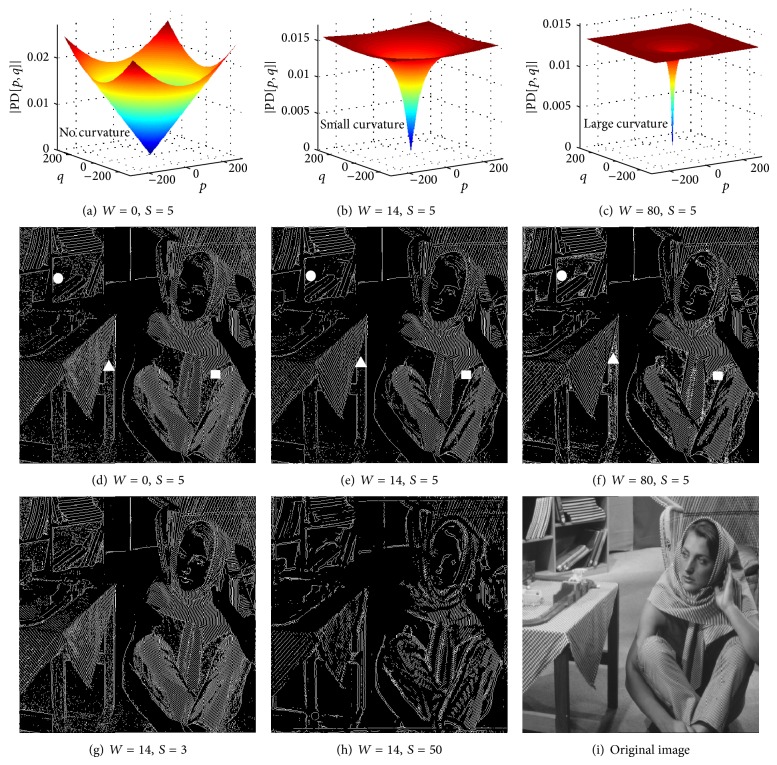
Effect of warp (*W*) and the strength (*S*) of phase applied to a sample image (shown in (i)) on the edge detection performance in the proposed method. (a) to (f) Comparison of edge detection results with three different amounts of *W* in the phase applied to the image. Phase derivative profiles are shown in (a) to (c) and the corresponding edge detection results are shown in (d) to (f). As seen, edge detection with medium warp has better noise performance than the case with very large warp or the case with linear phase derivative; compare regions indicated with white triangular, circle, and rectangular markers. (e), (g), and (h) Comparison of edge detection performance for the case of *W* = 14 with three different amounts of *S* applied to the image. As seen, larger phase results in less edge noises but also less resolution to detect the edges. Thus, there is an optimum value for *S* and *W* parameters to reduce the edge noises while preserving the vital edge information.

Figures 2(a) to 2(c) show typical phase derivative profiles that result in edge detection. The kernel applies a phase that increases with spatial frequency. Since edges contain higher frequencies, they are assigned a larger phase and therefore are spatially highlighted in the phase of the transformed image.

Parameters of the kernel (*S* and *W*) control the edge detection process. In the proposed method, there is a trade-off between spatial resolution and noises of edge detection. A larger phase (larger *S*) results in better noise performance in edge detection but at the expense of lower spatial resolution. Also a larger warp (larger *W*) in the phase derivative results in a sharper edge but it also increases the edge noise. These parameters can be adjusted manually or optimized by iterative algorithms. They can be either globally fixed or locally optimized.

Figure 2 illustrates the effect of warp and the strength of applied phase on the edge detection performance. The test image used here is the “Barbara” image shown in Figure 2(i). Figures 2(d) to 2(f) compare the edge detection results with different amounts of warp (*W*) in the applied phase. Figures 2(a) to 2(c) show the phase derivative (group delay) profiles and Figures 2(d) to 2(f) show the corresponding edge images. In all cases, identical localization kernel bandwidth (Δ*f* = 2), thresholding (Thresh = 0.047), and morphological operations are used. Three different amounts of warp are considered: very small warp (*W* = 0.001) corresponding to linear phase derivative (Figures 2(a) and 2(d)), medium warp (*W* = 14) (Figures 2(b) and 2(e)), and large warp (*W* = 80) (Figures 2(c) and 2(f)). In this paper, all the *S* and *W* parameters are calculated assuming the spatial span of image is from −0.5 to 0.5 in each of the two dimensions. Compared regions are indicated with white triangular, circle, and rectangular markers. As seen, edge detection with medium warp has better noise performance than the case with very large warp or the case with linear phase derivative. Figures 2(e), 2(g), and 2(h) compare the performance of edge detection for three different amounts of phase strength (*S*). In all cases, identical warp value of *W* = 14, localization kernel bandwidth (Δ*f* = 2), thresholding (Thresh = 0.047), and morphological operations are used. As seen, larger phase strength results in less noise but also less resolution for edge detection. To summarize, Figure 2 indicates that value for *S* and *W* parameters can be used to tune and optimize the edge detection performance.

Here we show qualitative comparison of edge detection using the proposed method with the powerful and popular Canny and Sobel methods. We emphasize that these comparisons are not intended as quantitative benchmarks but rather as a qualitative validation of the functionality of the new method proposed here.

**Figure 3:**
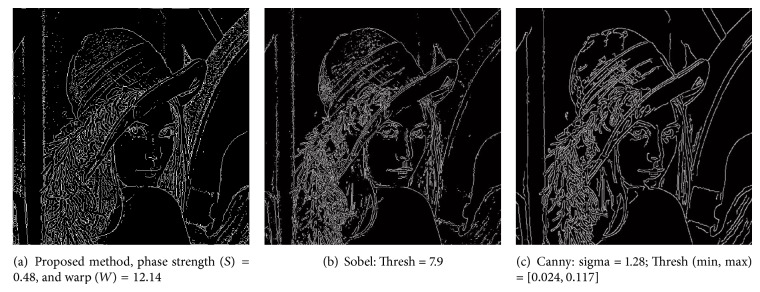
Qualitative comparison of the performance of the proposed method for edge detection (a) to the Sobel (b) and Canny (c) methods. Image under analysis is the “Lena” image with 512 × 512 pixels. For the proposed method, designed parameters are phase strength *S* = 0.48, phase warp *W* = 12.14, localization kernel bandwidth Δ*f* = 0.21, and binary threshold = 0.028. Designed threshold value for Sobel method is 7.9 and designed parameters for Canny method are sigma = 1.28, low threshold = 0.024, and high threshold = 0.117.

The image under analysis is a gray-scale Lena image with 512 × 512 pixels. Results of edge detection using the three methods are shown in Figure 3. Edge detection using the proposed method is shown in Figure 3(a). For the proposed method, designed parameters are phase strength *S* = 0.48, phase warp *W* = 12.14, localization kernel bandwidth Δ*f* = 0.21, and binary threshold = 0.028. Morphological operations used for the result shown in Figure 3(a) include edge thinning and isolated pixel removing. Edge detection using Sobel method with threshold value of 7.9 is shown in Figure 3(b). Edge detection using Canny method with sigma value of 1.28, low threshold values of 0.024, and high threshold value of 0.117 is shown in Figure 3(c). Sobel and Canny methods were implemented using the embedded functions in MATLAB software. All the three methods use postmorphological operations such as edge thinning and cleaning the isolated edge pixels. As evident in Figure 3, edges are accurately extracted with all three techniques. We note that, in a few regions (e.g., nose), Sobel and Canny provide more complete edge profile and have less edge noises. For the present technique, these issues can be improved by optimization of the PST kernel, localization kernel, and threshold settings.

## 3. Experimental Results

Here we show some examples of the proposed edge detection algorithm on biomedical images. In particular, we consider edge detection of histology and brain MRI images.

**Figure 4:**
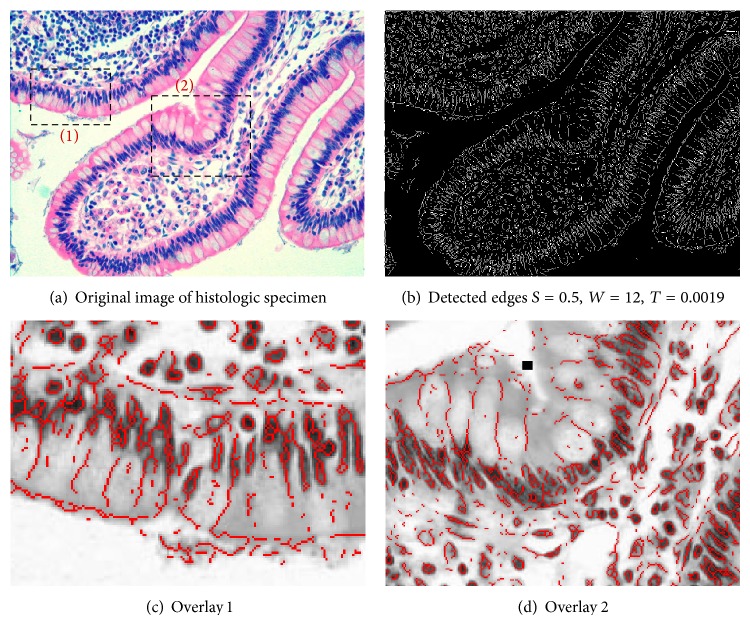
Edge detection of biomedical images based on Phase Stretch Transform (PST). In this example, image under analysis is histologic specimen of a tissue stained with hematoxylin and eosin. (a) Original image. (b) Detected edges using the proposed method. (c) and (d) Detected edges overlaid with the original image for the two boxes shown in (a). For the proposed method, designed parameters are phase strength *S* = 0.5, phase warp *W* = 12, localization kernel bandwidth Δ*f* = 0.21, and binary threshold = 0.0019. Figure 4 shows accurate edge detection using the proposed method. We also note that the weak edges shown in (d) with black square are not extracted properly. This can be improved by using better thresholding and localization methods.

In the first example, the image under analysis is a histologic specimen of a tissue stained with hematoxylin and eosin with 800 × 600 pixels; see Figure 4(a). Edge detection using the proposed method is shown in Figure 4(b). For the proposed method, designed parameters are phase strength *S* = 0.5, phase warp *W* = 12, localization kernel bandwidth Δ*f* = 0.21, and binary threshold = 0.0019. Morphological operations used for the result shown in Figure 4(b) include edge thinning and isolated pixel removing. We have also shown the detected edges overlaid with the gray-scale version of the original image in Figures 4(c) and 4(d). As evident in Figure 4, edges are accurately extracted using the proposed technique. We note that in a few regions with weaker edges (see Figure 4(d), the region around the black square) the edges are not extracted. For the present technique, these issues can be improved by optimization of the PST kernel, localization kernel, and threshold settings.

**Figure 5:**
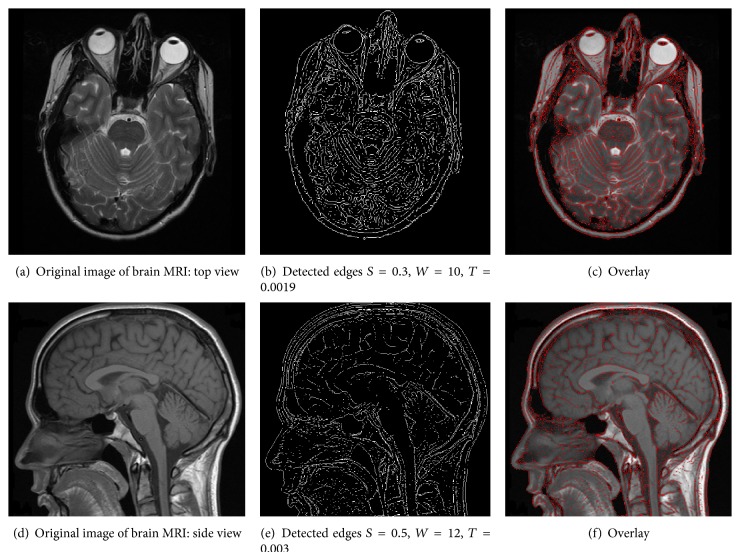
Edge detection of biomedical images based on Phase Stretch Transform (PST). In the two examples presented here, images under analysis are top and side view brain MRI images. (a) and (d) Original images. (b) and (e) Detected edges using the proposed method. (c) and (f) Detected edges overlaid with the original image. As evident in the figure, in both examples, the image edges are accurately extracted using the proposed method.

In the second example, the images under analysis are two gray-scale brain MRI images: (i) view from the top and (ii) view from the side. The top view brain MRI image has 500 × 500 pixels and is shown in Figure 5(a). The side view brain MRI image has 652 × 600 pixels and is shown in Figure 5(d). Edge detection using the proposed method for the two sample images is shown in Figures 5(b) and 5(e). We have also shown the detected edges overlaid with the gray-scale version of the original image in Figures 5(c) and 5(f). Designed edge detection parameters for the top view brain MRI image are phase strength *S* = 0.3, phase warp *W* = 10, localization kernel bandwidth Δ*f* = 0.21, and binary threshold = 0.0019. For the side view brain MRI image case, the designed edge detection parameters are phase strength *S* = 0.5, phase warp *W* = 12, localization kernel bandwidth Δ*f* = 0.21, and binary threshold = 0.003. In both cases, edge thinning and isolated pixel removing were used for morphological operations.  Figure 5 proves that edges are accurately extracted using the proposed technique for the two examples of brain MRI images as well.

## 4. Conclusions

A new approach to edge detection in images has been introduced. It is based on a nonlinear dispersive phase operation applied to the image. The output phase of the transform reveals transitions in image intensity and can be used for edge detection and feature extraction. Examples of edge detection on biomedical images presented here show that the proposed edge detection algorithm has promising application in segmentation and analysis of biomedical images.
